# Seventeenth Annual Meeting of the Mississippi Valley Association

**Published:** 1861-04

**Authors:** 


### Proceedings of Societies.

### SEVENTEENTH ANNUAL MEETING OF THE MISSISSIPPI VALLEY ASSOCIATION.

### DISCUSSIONS.

Dr. Atkinson read a paper entitled "*Plates over Fangs;*" and remarks being called for,

Dr. Taylor said that the practice must be varied to suit the cases. When some of the fangs were diseased, and some healthy and firm, he would generally extract all.

Dr. Atkinson responded that, if they were alternately healthy and diseased, he, too, would extract all.

Dr. Watt said the constitution of the patient must be taken into account—his physiology and pathology must both be carefully considered. In some constitutions, even when the mouth is in a comparatively healthy state, the roots can not be kept healthy, after the loss of the pulps. He referred to cases to show that, when the vital powers are low, plates over fangs will seldom prove satisfactory.

Dr. Taft remarked that the paper gives the prominent arguments for the retention of the fangs. The features are much better preserved, where they can be retained. The soft parts *will* change when they are removed. If there were but one or two fangs in the arch, and the force in mastication did not bear on them, he would remove them, even if healthy, as otherwise, there would likely be sufficient shrinkage around them to cause the plate to rock. If there were many in the arch, and they were so healthy that the pressure of mastication could be distributed evenly over them, he would leave them in. And, in such cases, perfect adaptation of the plate is very important. The margin of the stump should not be left sharp, or it would irritate the overlapping portion of the gum; and the plate should be adapted so as to rest firmly on the stump.

Dr. RICHARDSON inquired how it is that there is greater depression just below the alæ of the nose than elsewhere. He suggested that we may be mistaken in this matter. Was it not possible that the greater shrinkage here is only relative? The shortness of artificial gums rendered it impracticable to restore the features at this point; and the lip might be made too prominent below, while above, it might not appear sufficiently so.

### REGULAR DISCUSSIONS.

### PROFESSIONAL ETHICS.

Dr. RICHARDSON remarked that it was common for one dentist to give his opinion of an operation performed by another, by request, or otherwise. He thought it was sometimes proper to give such opinion; but great caution should be exercised. He would confess himself quite sensitive in this respect. He often had to fill teeth, and, doubtless others had, when not in proper condition, or, when the patient was not in proper condition to make perfect operations. He gave in illustration the case of a little boy, a relative, who was exceedingly nervous, and as he could not control him, he had to fill under water, and the filling he regarded as imperfect—thought it would not save the tooth beyond two or three years. He did the best he could under the circumstances, and would refill as soon as he could control the patient. Now, if this patient should pass into other hands, this filling might be criticised, without a knowledge of the circumstances, and his operations might be condemned on account of it. It was not possible to pronounce sentence in such cases without doing injustice.

Dr M'CULLUM said that nothing gave him so much satisfaction as to see, and to have the opportunity to speak well of another's operations. Even when work was defective, the difficulty was often the result of a slight oversight. The profession, he said, should cultivate a spirit of sociality. For his own part, he preferred the society of dentists to that of other men.

Dr. FOOTE said that the first consideration is to be honest. If he saw work badly—dishonestly done, he felt it to be his duty to denounce it. He recently saw an amalgam filling with a gold clasp lying against it. The patient had suffered in consequence, for years, with nervousness—had a horror of wearing the plate,—had called on the operator again, but instead of removing the cause, he merely lanced the gums. In this case the patient's health had been impaired; and he could not help but express his disapprobation. On the other hand, he had recently seen a very good, finely-finished gold filling in the mouth of a child, which was brought to him on account of a little new decay near the filling. He took great pleasure in commending the filling, and in informing the mother that the present decay was not on account of any defect in work of the former operator.

Dr. M'CLELLAND said it was a common thing for us to see the operations of other dentists; and we should never express our feelings or opinions prematurely. We often had to remove the fillings of others, on account of toothache or other causes; and we should be acquainted with, and consider all the history of the case, before giving an opinion. And to avoid the unjust criticisms of other operators, we should be always honest and candid when we do the work. When the circumstances were not such as would allow him to do such filling as he would like, he was in the habit of telling his patients so—tell them that the filling is temporary—will need renewing, but that it is the best you can do under the circumstances. In a general way, those who do the poorest work, he said, were the loudest in condemnation of that done by others. He stated, in conclusion, that we have among us far too much professional jealousy.

Dr. Atkinson said that a man who is a man needs no instruction in regard to these matters; but professional courtesy is not exercised as it should be, even among clergymen; and the same is true in regard to the professions of law and medicine; and it is, therefore, not to be wondered at that our profession—a mere boy—should prove deficient. He would suppose a case for illustration—a case of frequent occurrence, too,—in which a patient calls who has had teeth filled by another operator, and the work warranted—which was the first mistake in the matter—but the work has failed, and though repeatedly refilled, is still a failure—it would still be our duty to advise the patient, as a general rule, to return and give the operator a chance to make good his work; but if the patient is unwilling to trust him farther with the case, and if you know he is incompetent, it is your duty to do the best you can for the case; and it would be much less embarrassing if you did not know who had made the failure. If the patient has met his obligations to the former operator, we are at full liberty under such circumstances, to operate for him.

Jealousy is the offspring of ignorance, and is the result of an undue desire on the part of its subject, to pass for more than he is worth.

### STRUCTURE AND NUTRITION OF DENTAL TISSUES.

Dr. Atkinson remarked that nutrition is always extravascular, and is the same in dentine as in other tissues. The nutritive material transudes from the vessels through the pulp-membrane. He did not agree with Tomes in regard to the passage of nerve fibrils through this membrane into the dentine. In cases of persons dying by violence, where the circulation was very vigorous, coagulable lymph might be formed in the tubules, and Mr. Tomes may have been thus deceived. Nerves, he said, do not terminate in sharp points, but in loops, or by passing into other nerves. No holes are discovered in the pulp-membrane, by examination with the microscope, and he inferred from that, that no vessels pass through it to the dentine. The circulation through dentine he regarded as similar to that of the vegetable kingdom.

REPORTS OF CASES—*Written or Verbal.*

Dr. ATKINSON, in answer to an inquiry, gave it as his opinion that he had never seen a case of neuralgia, that was not, to some extent, under miasmatic influence.

He reported a case of two alveolar abscesses, one of which he thought was matured, and cut into it, but found it was not ready. He applied fomentations, nauseated and narcotised the patient, but the cut place did not suppurate, though the abscess pointed at another place. The other matured, was opened and treated, and it healed kindly. He used tonics to counteract miasmatic influence, and to invigorate the general system.

Dr. TAFT referred to a case of diseased antrum, of twelve months' standing, with a discharge from the right nostril, not very fetid, but at times very copious. The antrum was often painful, but sometimes not. There was no swelling, no soreness to the touch, and no tooth apparently much diseased. A decayed molar was removed, and the cavity of the antrum opened through the socket, but very little pus escaped. The floor of the antrum was tender to the touch, and there was great thickening of the lining membrane, but no tumor. He washed out the cavity with soap and water, injected hyperchloride of soda into it, and prescribed invigorating constitutional treatment. He afterward injected a solution of creosote, and again tincture of myrrh and arnica; but there was no improvement in the case, and the discharge of matter rather increased. The orifice was kept open by the insertion of a piece of pine wood. He had not seen the case for a month or two, as the patient resides in a distant part of the State, but was informed that it was growing worse, that the general health was failing, and that the left antrum was now in a worse condition than the right was when he first saw it. The case had been treated by a number of physicians. He wished to hear suggestions from others. (Here the remarks and suggestions became so much like social conversation, that we listened intently, and forgot to report.—W.)

Dr. PEASE spoke of a case in which there was considerable swelling, from disease of the antrum. A lateral incisor pivot tooth was worn. He took off the artificial crown, and enlarged the opening through the apex of the root, which was followed by a free discharge of pus. He now removed the root, and found exposed bone in the antrum. He resorted to the usual remedies, and inserted a silver tube, with a flange, to allow the pus to escape freely. He prescribed iodide of potassium and sarsaparilla, and injected, occasionally, a solution of creosote. The opening began to fill, and progressed gradually, till it is now well. It was under treatment about a year.

*Another Case.*—The nerve of a carious molar was destroyed by another dentist, and the cavity of decay filled. The result was an offensive discharge from the antrum into the nose. He took out the filling, treated the canals, filled them and the tooth, and all is well.

*Another Case.*—Neuralgia of long standing. The patient had resorted to a variety of treatment. Had spent a portion of the past summer at "the springs;" but the disease continued. In conversation with the patient's sister, recently, he had told her that neuralgia belongs to dentists, rather than to physicians. The patient called on him, and he destroyed the pulp of a wisdom tooth, and there was perfect relief.

Dr. Atkinson remarked that in a case like that reported by Dr. Taft, he would use, as a local application, pure creosote once a week, or possibly a little oftener. He thought escharotics were often used too frequently, especially in feeble constitutions.

Dr. Pease remarked that he did not like the action of nitrate of silver on exposed bone.

Dr. Chapman (Physician) remarked that the use of escharotics was an important question. The impression was far too common that to use the medicine was about all that was necessary. There was too often a lack of due discrimination. He was pleased to see that such was not the case here. He remarked that when a patient is far advanced in life, violent escharotics are not applicable, as the vital powers are too feeble to make the necessary resistance, or to make due repair. Even some young persons have correspondingly feeble constitutions, when powerful escharotics will do harm. Now, it is very important to use the right escharotic, and to have it of the proper strength. He had used creosote and nitrate of silver in disease of the pharynx. Had succeeded in a recent case, by occasional applications, though the ulcerations were very numerous, while a former physician had failed, by applying the nitrate every day, or oftener.

Dr. Lyman reported a case of diseased antrum, of five years' standing. The mucous membrane was thickened, and there was a greenish-yellow discharge from the nose. He drilled through the root of the eye tooth, and passed a broach, two inches long, through it, without meeting with any resistance. He annealed the broach, and made a hook on the end of it, and endeavored with it to destroy the sac. He dipped the broach in creosote, and re-inserted it, and, on removing it, it was covered with "curdled" matter. He pumped creosote through the tooth, till it was felt in the nose. At this stage a physician was called in, and it was determined to extract the tooth. The cavity of the antrum was afterward washed out with warm water, and dressed with tannin and glycerine, and the recovery appears to be perfect.

Dr. M'Clelland referred to a case in which a molar tooth, with the pulp exposed, had been filled, after capping the nerve. The pulp died, and an abscess was the result. The disease extended to the antrum, and the discharge through the nose was very great, but became less copious, without treatment. Redness appeared on the cheek, and the tooth was extracted. There was now a discharge over the back part of the palate, which, however, became less and less in quantity, till it entirely disappeared. There is still pain in the region of the antrum, but not of a neuralgic character, and now he wished to know how to relieve it.

Dr. Atkinson said he would open the antrum, and inject tincture of iodine, one part, and water, two parts. This, he thought, would have a better effect than direct escharotics.

Dr. Baxter, when at a distance from home, had extracted a second bicuspid, on account of diseased antrum, and opened the antrum, through the socket, with a trocar, and directed the patient to blow a solution of common salt through it. The patient got well in four weeks.

Dr. Atkinson remarked that while the natural opening into the nostril remained pervious, the cases were not so difficult to treat.

Dr. Taft said there were notices in some of the French journals of what was claimed as a newly discovered disease, called by them, "*Expulsive Gengivitis.*" He had seen a number of cases of this disease. Many years ago, a patient came into his hands, whose teeth became loose, protruded, and seemed to be expelled from their sockets, independent of depositions of tartar, or other apparent local causes. The front teeth protruded so much that their cutting edges looked directly forward. They were removed, and the disease manifested itself about the cuspids, and, afterward, the bicuspids. The vital powers were feeble; and the patient has since died. In all the cases observed by him, the margins of the gums appeared to have less than their normal supply of blood.

Dr. Watt had seen similar cases, and remembered the one specially described by Dr. Taft. In the treatment of the disease, he thought constitutional remedies would be required; and local treatment should not be such as would produce much irritation. He had not made the desired analysis, but presumed there would, ordinarily, be found an alkaline reaction in the secretions. If so, the use of vegetable or mineral acids would be indicated.

Dr. ATKINSON said he thought the ordinary cases of diseased gums were scorbutic, and could be profitably treated, locally, with acetic acid and chlorate of potash; but this matter was new to him. He had never seen a case like the one described by Dr. Taft.

Dr. BAXTER had seen such cases. In some he had seen alternate teeth affected. He had found the disease more common in the black and mixed races, than among the whites. He had relieved some cases by cutting down the gum, and washing with whisky and sugar.

Dr. PEASE was not certain that he recognized the disease from the descriptions given. He would refer to a case of ordinary gum disease. The gum was receding from a single tooth, but he could not discover that there was much pus discharged from it. He had the patient use iodide of potassium and chlorate of potash, internally, and painted the gum with tincture of iodine, and it got well.

Dr. ATKINSON reported the case of a lady in whose mouth the outer wall of the alveolar processes was necrosed from the left lateral incisor to the right canine. There were, also, three fistulous openings. He took off the necrosed portion, and he was satisfied that there was not more than two-thirds of the peridental membrane alive. The teeth were, of course, very loose. He inserted wedges between them, to distribute the force equally, and filled those which required filling, forming inclined planes on their lingual faces, to press the teeth outward to their proper positions. New process formed over the central incisor, thicker than the old; and the tooth stood firmer than before it became diseased.

*Another Case.*—The four incisors had been filled, and the nerves died, and the teeth were much discolored; and three abscesses were discharging from their roots. He removed small portions of necrosed bone, after which the abscesses yielded to ordinary treatment, and the teeth are now tight and firm.

*Another Case.*—Arsenious acid had been used in a first lower molar. The transverse portion of the alveolar process died, and was removed, and new bone formed in its place.

Dr. C. F. KNOWLTON mentioned the case of a young lady, twenty-five years old, of good constitution, and having healthy gums. A year ago one of the upper central incisors began to protrude. At the end of six months, it stood outward at an angle of about forty-five degrees. The rest were all regular and solid.

Dr. M'CLELLAND had seen the incisors protrude when the wisdom teeth were making their appearance.

Dr. LYMAN thought there was probably a mechanical cause. He had seen the front teeth forced outward by the patient sucking the thumb.

Dr. PEASE had seen a case somewhat similar to Dr. Knowlton's; but the pulp was dead. He cut off the crown and inserted a pivot tooth. A year after, the pivot tooth protruded, the case was treated variously for a year or two, but finally the tooth was extracted.

Dr. C. F. KNOWLTON referred to a case of diseased antrum, which occurred long ago. The second bicuspid was extracted. The next day the face was much swollen and red. The anterior bicuspid had been broken off before. He drilled through it, and there was a free discharge of pus, but the orifice soon closed up. A physician was called in, who said the first molar must be extracted—that the fangs of bicuspids never interfered with the antrum. He purged, bathed, leeched, and applied stramonium leaves, and left, but soon returned, as he said he had put the leaves on wrong side up, and the consequences might have been fatal, if he had allowed them to remain. At this return, the physician insisted that the first molar must be removed, and it was done, under protest; but no relief was obtained. He now re-opened the antrum, washed it out with soap and water, and injected diluted creosote, ten drops to the hundred. As the case got better, the other side became affected in the same way; and the case passed out of his observation.

Dr. M'Clelland spoke of a soft tumor on the gum, which had continued several weeks without pain. He advised the extraction of the adjacent tooth; but the patient would not consent. He opened the tumor, and it discharged a dull, milky fluid, and sunk down, but in three minutes was as full as ever. He did not get leave to re-open it. He would like to know where the fluid came from.

### FILLING TEETH.

Dr. Richardson detailed his method of filling crown cavities in molars. He was partial to welded plugs, but did not, as some, begin at the bottom of the cavity, and weld small pellets all the way up. He tried to fill with as little labor as would make perfect work. He formed cylinders by the usual mode, cutting his strips about half as wide as the cavity is deep, and made the first one as large as he could conveniently introduce into the cavity. He condensed this by lateral pressure, and introduced smaller cylinders, and in this way he filled the cavity about half full, with non-adhesive foil as a basement filling. To this he welded adhesive gold in strips, with serrated points, and finished with small pellets cut from the strip.

In an approximate cavity, in a lower bicuspid, for example, when the decay extends a little below the gum, he inserts a block, and lets the end project over the margin of the gum, and uses smaller blocks above this, and welds in as usual.

Dr. Atkinson commonly uses the mallet in filling. In approximate decays the grinding surface should not be unnecessarily destroyed. He detailed his mode of filling a posterior approximate decay in a lower bicuspid. The tooth should be supported by wedges between it and the adjacent teeth. He would make little cylindrical pits in the cervical wall, for retaining points, and fill these carefully. Then he would weld a pellet of gold to one of them, and hold it there with an instrument, or have his assistant do it, while he welded to the other point. He would build against the walls and finish perfectly on the surface. If the surface was made perfect, it did not matter if the center was porous.

Dr. WELLS described his method of filling a certain kind of cavity, illustrating by diagrams on the blackboard. His illustrations were clear and his manipulations ingenious, but we can not do justice to them without diagrams. (We would be pleased to hear from him at length in the Register.—W.)

A general exchange of views in regard to the use of the mallet here took place, and as the hour for adjournment had arrived, the remaining subjects were postponed.

Dr. M'Clelland exhibited a new instrument for holding the lips apart while taking impressions. He calls it an "Impression Fork." Dr. Lyman exhibited an elastic mallet, for use in plugging. Dr. Pease introduced an ingenious and efficient little instrument, for holding a napkin between the lower gum and the lips. J. T. Toland exhibited a new vulcanizer, and other apparatus. The members lingered as if reluctant to leave; and though not as full a meeting as sometimes, it was both pleasant and profitable. W.

---

ANTI-TOBACCO MOVEMENT.—A strong movement in opposition to the present extensive use of tobacco has been commenced in Edinburgh. At a late meeting, the following resolution was adopted: "That as smoking has a tendency to encourage the drinking usages of society, not only by creating morbid thirst, but also by its exhausting power, thereby inducing recourse to a falsely supposed substitute, it is greatly calculated to foster crime and dissipation in the masses."

